# Biomarkers to guide antibiotic timing and administration in infected patients presenting to the emergency department

**DOI:** 10.1186/s13054-019-2422-9

**Published:** 2019-04-25

**Authors:** Mari Rosenqvist, Darius Cameron Wilson, Lena Tegnér, Maria Bengtsson-Toni, Marjaneh Peyman, Juan Gonzalez del Castillo, Kordo Saeed, Olle Melander

**Affiliations:** 1 Infectious Disease Unit, Skåne University Hospital, Department of Clinical Sciences Malmö, Lund University, Malmö, Sweden; 2 B·R·A·H·M·S GmbH, Hennigsdorf, Germany; 3 Emergency Department, Instituto de Investigación Sanitaria (IdISSC), Hospital Clínico San Carlos, Madrid, Spain; 4 Department of Microbiology, Hampshire Hospitals NHS Foundation Trust, Winchester and Basingstoke, UK; 5 Department of Internal Medicine, Skåne University Hospital, Malmö, Sweden

Antibiotics are often prescribed in the emergency department (ED) to patients presenting with a suspected infection before any definitive diagnosis can be made [1]. However, increasing antibiotic resistance and detrimental effects on the microbiota require their use to be limited to those with a high likelihood of bacterial infection or the potential for further clinical deterioration. Conversely, withheld or delayed treatment in higher severity patients may lead to increased morbidity and mortality rates [2]. Thus, an accurate assessment of antibiotic requirement and speed of administration is crucial.

Current tools to aid clinical decision-making include the use of procalcitonin (PCT) and C-reactive protein (CRP). However, recent interventional evidence in the ED has shown few differences between conventional biomarker-guided therapy and standard practice [1, 3], despite protocol compliance, patient selection and cut-off concerns. This post hoc analysis of a patient subset (Malmö, Sweden) from our previous investigation [4] compared the use of PCT, CRP and lactate to the novel biomarker mid-regional proadrenomedullin (MR-proADM) in guiding antibiotic administration during treatment within the ED.

Within this subset (*N* = 213), 26 (12.2%), patients were prescribed antibiotics < 48 h prior to presentation, whilst 187 (87.8%) were administered antibiotics during ED assessment. Of these patients, 164 (77.0%) were treated with intravenous (i.v.) and 23 (10.8%) with oral antibiotics. The median time to initial administration was 93 [28–160] min, with 71 (43.8%) patients receiving therapy within 60 min. Univariate and multivariate logistic regression found that MR-proADM had the strongest association with the requirement for antibiotic administration during ED treatment (Table 1). Interestingly, MR-proADM (Spearman *ρ* = − 0.31, *p* < 0.001) and lactate (Spearman *ρ* = − 0.25, *p* = 0.002) were the only parameters to be significantly negatively correlated with the time to antibiotic administration, with significant differences found at optimised MR-proADM cut-offs for antibiotic administration (1.27 nmol/L: 139 [76–211] vs 43 [26–135] min; *p* < 0.001) or pre-established [4] cut-offs for mortality prediction (1.54 nmol/L: 124 [33–199] vs 42 [26–122] min; *p* = 0.002). Similar results were also found for MR-proADM within previously established PCT concentration ranges [5] (Table 2), with an absence of ICU admission or 28-day mortality in patients with low MR-proADM concentrations, despite lower antibiotic administration rates and a significantly longer time to administration.

**Table 1:** Univariate and Multivariate analyses found that MR-proADM had the strongest correlation with the requirement for antibiotic administration during ED treatment

Biomarker	Patient population (*N*)	Antibiotic administration (*N*)	*p* value	C index	Univariate OR [95% CI]	Multivariate OR [95% CI]
MR-proADM	213	164	< 0.001	0.76	3.1 [1.9–4.9]	3.3 [1.9–5.9]
PCT	213	164	< 0.001	0.74	2.7 [1.7–4.3]	2.7 [1.7–4.5]
CRP	207	159	< 0.001	0.68	1.8 [1.3–2.5]	1.9 [1.4–2.8]
Lactate	204	158	0.002	0.66	1.8 [1.2–2.6]	1.6 [1.1–2.5]

Age, cardiovascular, neurological, renal and malignancy comorbidities were used as adjusting variables within the multivariate regression analysis, as previously outlined [4]. Univariate and multivariate odds ratios were expressed per 1 SD increment of the log-transformed value for each respective biomarker. *CI* confidence interval, *CRP* C-reactive protein, *DF* degrees of freedom, *MR-proADM* mid-regional proadrenomedullin, *N* number, *OR* odds ratio, *PCT* procalcitonin

**Table 2:** Low MR-proADM concentrations resulted in an absence of ICU admission or 28-day mortality, despite lower antibiotic administration rates and a significantly longer time to administration, irrespective of corresponding PCT concentration

Patient subgroups	MR-proADM concentration	
	< 1.27 (nmol/L)	≥ 1.27 (nmol/L)
Subgroup 1: PCT concentration: < 0.25 μg/L (*N* = 106)		
Patients (*N*)	65	41
Antibiotic administration (*N*, %)	35 (53.8%)	34 (82.9%)
Time to antibiotic administration (min) (median, Q1-Q3)	127 [45.0–220]	42 [25.8–116]
Composite of 28-day mortality and ICU admission (*N*, %)	0 (0.0%)	7 (17.1%)
Subgroup 2: PCT concentration: ≥ 0.25 and < 0.50 μg/L (*N* = 24)		
Patients (*N*)	8	16
Antibiotic administration (*N*, %)	7 (87.5%)	15 (93.8%)
Time to antibiotic administration (min) (median, Q1–Q3)	165 [88–305]	50 [19.3–186]
Composite of 28-day mortality and ICU admission (*N*, %)	0 (0.0%)	1 (6.3%)
Subgroup 3: PCT concentration: ≥ 0.50 μg/L (*N* = 83)		
Patients (*N*)	21	62
Antibiotic administration (*N*, %)	15 (71.4%)	59 (95.2%)
Time to antibiotic administration (min) (median, Q1–Q3)	131 [92.8–166]	45 [26–136.5]
Composite of 28-day mortality and ICU admission (*N*, %)	0 (0.0%)	15 (24.2%)

*MR-proADM* mid-regional proadrenomedullin, *N* number, *PCT* procalcitonin, *Q* quartile

Results suggest that delayed antibiotic administration in patients with low MR-proADM concentrations may result in few adverse effects, potentially allowing for a more detailed clinical assessment prior to any subsequent initiation. Further studies in larger patient populations are required to confirm these initial findings.

## Abbreviations

CI: Confidence interval
CRP: C-reactive protein
ED: Emergency department
i.v.: Intravenous
ICU: Intensive care unit
MR-proADM: Mid-regional proadrenomedullin
N: Number
OR: Odds ratio
PCT: Procalcitonin
